# Internal jugular venous flow responses to graded lower body negative pressure: a sex-stratified analysis

**DOI:** 10.3389/fphys.2026.1873225

**Published:** 2026-09-15

**Authors:** Andrej Bergauer, Janez Urevc, Bianca Steuber, Karin Schmid-Zalaudek, Vid Pivec, Nandu Goswami

**Affiliations:** 1 Division of Physiology, Otto Loewi Research Center of Vascular Biology, Immunity and Inflammation, Medical University of Graz, Graz, Austria; 2 Department of Surgery, General Hospital (LKH Südweststeiermark), Wagna, Austria; 3 University of Ljubljana, Faculty of Mechanical Engineering, University of Ljubljana, Ljubljana, Slovenia; 4 Center for Space and Aviation Health, Mohammed Bin Rashid University of Medicine and Health Sciences, Dubai, United Arab Emirates

**Keywords:** cardiovascular physiology, central hypovolemia, internal jugular vein, jugular venous flow, jugular venous outflow, lower body negative pressure, sex-stratified analysis, ultrasound

## Abstract

**Background:**

The internal jugular vein (IJV) is the primary pathway for cerebral venous drainage in the supine position. Although lower body negative pressure (LBNP) is widely used to simulate central hypovolemia, the dose–response relationship between graded LBNP and jugular venous flow, and potential sex differences therein, remain incompletely characterized. We hypothesized that graded LBNP would produce a progressive, dose-dependent reduction in jugular venous flow, and examined whether this response differed between sexes.

**Methods:**

Forty-four healthy young adults (22 female, 22 male) underwent graded LBNP (−10 to −40 mmHg) in the supine position. IJV cross-sectional area and blood flow velocity were assessed using B-mode ultrasound and pulsed-wave Doppler at the mid-neck (J2 segment). Volumetric flow was calculated as the product of mean area and mean envelope velocity. Relative flow changes (normalized to individual supine baseline) were log-ratio transformed prior to analysis. Data were analyzed using linear mixed-effects models with state, sex, and their interaction as fixed effects.

**Results:**

Female participants showed significant jugular venous flow reductions at LBNP30 and LBNP40 (bothp< 0.001); males showed significant reductions from LBNP20 onward (p ≤0.0004). The main effect of sex was not significant (p = 1.00), and no state× sex interaction reached significance across LBNP levels or during recovery. A non-significant trend toward a larger flow reduction in females at LBNP40 (p = 0.056) did not persist in a completers-only sensitivity analysis (p = 0.16). Baseline IJV cross-sectional area was larger in males (p = 0.031) but did not differ after indexing to body surface area (p = 0.23).

**Conclusion:**

Graded LBNP induces a progressive, dose-dependent reduction in jugular venous flow from moderate levels onward. Despite sex differences in Systemic responses, the jugular venous flow response was broadly similar between females and males, and baseline morphological differences were largely Attributable to body size. These observations are consistent with jugular venous outflow during central hypovolemia being governed predominantly by mechanical factors, although the modest sample size and exploratory analyses warrant confirmation. These findings inform the physiological understanding of jugular venous responses to central hypovolemia and are relevant to spaceflight countermeasures.

## 1 Introduction

The internal jugular vein (IJV) is the primary extracranial pathway for cerebral venous outflow and plays a key role in regulating intracranial venous pressure and cerebral hemodynamics. Owing to its high compliance and direct anatomical continuity with the thoracic venous system, the IJV is highly sensitive to changes in central blood volume and transmural pressure. In the supine position, the IJVs remain distended and constitute the dominant route of cerebral venous drainage. In contrast, during upright posture, partial jugular collapse redirects venous outflow toward the vertebral venous plexus, altering cerebral venous drainage patterns (Gisolf et al., 2004). Accordingly, changes in IJV geometry and blood flow provide a sensitive, non-invasive index of central venous loading and cerebral venous drainage in both physiological and pathophysiological conditions (Chua Chiaco et al., 2013; Kenny et al., 2025).

Lower body negative pressure (LBNP) is an established experimental model for inducing graded central hypovolemia by redistributing blood volume from the thoracic compartment toward the lower extremities. Extensive validation work has demonstrated that progressive LBNP produces central blood volume reductions comparable to controlled hemorrhage, with −20 to −30 mmHg corresponding to approximately 400–600 mL of pooled blood and −40 mmHg corresponding to ∼700–1,000 mL or more, representing 10–20% of total blood volume (Cooke et al., 2004; Wolthuis et al., 1974; Hinojosa-Laborde et al., 2014; Goswami et al., 2019a; Goswami et al., 2019b). At these levels, LBNP approaches the physiological boundary associated with cardiovascular decompensation while remaining a reproducible and reversible experimental stimulus in healthy participants.

When applied in the supine position, LBNP reduces central venous return without direct gravitational compression of the neck veins, resulting in smaller reductions in IJV cross-sectional area than those observed during head-up tilt, where hydrostatic effects dominate (Ogoh et al., 2022). Nonetheless, −20 to −40 mmHg LBNP produces central blood volume shifts and reductions in cardiac output comparable to upright standing or 60^°^ head-up tilt (Taneja et al., 2007). This distinction allows isolation of the effects of reduced central blood volume and thoracic pressure gradients on jugular venous hemodynamics, independent of postural hydrostatic forces. While arterial pressure regulation and autonomic reflexes during LBNP are well characterized, cerebral venous outflow responses to progressive central hypovolemia remain comparatively less studied (Pawelczyk et al., 2001; Nette et al., 2003; Goswami, 2023).

Interest in jugular venous physiology has increased further in the context of spaceflight, where the absence of a hydrostatic gradient leads to sustained cephalad fluid shifts, jugular vein distension, and altered venous flow patterns. These adaptations have been implicated in spaceflight-associated neuro-ocular syndrome and venous flow abnormalities observed during microgravity exposure (Mader et al., 2011; Lee et al., 2020; Harris et al., 2022; Blaber et al., 2011). The identification of IJV thrombosis and venous stasis during spaceflight has underscored the importance of understanding factors governing cerebral venous drainage and its modulation by countermeasures such as LBNP, which has been shown to reduce IJV volume and partially restore venous outflow in ground-based analogs and in-flight studies (Auñón-Chancellor et al., 2020; Marshall-Goebel et al., 2019; Arbeille et al., 2021; Goswami et al., 2019c, 2026, 2012).

Despite these advances, the dose–response relationship between graded central hypovolemia and jugular venous flow remains incompletely defined. Moreover, although sex differences in autonomic regulation and orthostatic tolerance are well documented, with females generally exhibiting lower tolerance to central hypovolemia, the extent to which these differences translate to sex-specific regulation of cerebral venous outflow is unclear (Convertino, 1998; Evans et al., 2001; Shankhwar et al., 2023; Goswami et al., 2015; Evans et al., 2015, 2018; Patel et al., 2016; Sachse et al., 2019).

Therefore, the primary aim of this study was to quantify changes in IJV morphology and blood flow during graded LBNP-induced central hypovolemia in healthy young adults using synchronized ultrasound imaging and beat-to-beat cardiovascular monitoring. Because the study was conducted in the supine position, in which the internal jugular veins remain distended and constitute the dominant route of cerebral venous drainage (Gisolf et al., 2004), the IJV was selected as the primary vessel of interest; the vertebral venous plexus, which assumes a larger drainage role during upright posture, was not assessed. A secondary aim was to determine whether these responses differ between males and females. We hypothesized that graded LBNP would produce a progressive, dose-dependent reduction in jugular venous flow, consistent with reduced central venous filling pressure and transmural pressure across the vein (Goswami et al., 2013).

The present study reports IJV hemodynamic data collected as part of the same experimental protocol and participant cohort described by Shankhwar et al. (2023). That study reported the systemic cardiovascular and autonomic responses of this cohort (heart rate, arterial blood pressure, stroke index, cardiac index, total peripheral resistance index, and heart-rate-variability indices), whereas the internal jugular vein morphology, blood velocity, and volumetric flow data acquired during the same experimental sessions were not reported therein and are analyzed here for the first time. Sex-specific responses to central hypovolemia across different phases of the menstrual cycle in the same cohort have been examined separately by Shankhwar et al. (2024).

## 2 Methods

The study was conducted at the Division of Physiology, Medical University of Graz, Austria. Ethical approval was obtained from the institutional ethics committee (Ref. EK 25–551 ex 12/13), and all procedures conformed to the Declaration of Helsinki. All participants received a detailed explanation of the study protocol and provided written informed consent prior to inclusion.

Details of participant recruitment and the experimental protocol have been described previously by Shankhwar et al. (2023). Briefly, young, healthy volunteers aged 18–30 years were recruited. Inclusion criteria comprised non-smoking status and absence of known cardiovascular, thrombotic, neurological, or psychological disorders; female participants were not pregnant at the time of testing. Participants were instructed to refrain from endurance exercise for 48 h and from caffeine consumption for 24 h prior to the experiment.

A total of 44 individuals were enrolled in the study. Baseline characteristics are reported for participants with valid data for the respective outcome (**Table 1**).

**Table 1:** Participant characteristics (analytical sample).

Variable	Sex	Mean ± SD	n	p-value (M vs F)
Age [y]	Female	22.1 ± 2.4	18	0.012
	Male	24.5 ± 3.3	20	
Height [cm]	Female	166.3 ± 4.7	18	<0.0001
	Male	180.9 ± 7.5	20	
Body mass [kg]	Female	57.2 ± 5.1	18	<0.0001
	Male	74.7 ± 9.8	20	
Body surface area [m^2^]	Female	1.63 ± 0.09	18	<0.0001
	Male	1.94 ± 0.15	20	

Female *n* = 18, Male *n* = 20. Values are mean ± SD for participants with valid baseline data.

Data are mean ± SD. p-values from Welch’s t-test (two-tailed). The analytical sample includes participants with valid baseline measurements for the respective variables. The total number of enrolled participants was 44; exclusions were due to insufficient ultrasound image quality or missing data.

Handling of missing and excluded data. Six participants were excluded from the analysis of jugular venous flow due to insufficient ultrasound image quality or missing key data precluding reliable quantification. In addition, seven participants did not complete the full LBNP protocol due to presyncopal symptoms; however, these participants were retained in the analysis, and all available measurements were included. Observations from uncompleted experimental conditions were treated as missing. Consequently, the effective sample size varies depending on the outcome variable and availability of valid measurements. Linear mixed-effects models allow inclusion of participants with incomplete measurements without listwise deletion.

### 2.1 Study design and LBNP protocol

All experiments were performed in a quiet, temperature-controlled laboratory (23–25^°^C, 50–55% relative humidity) between 09:00 and 13:00 to minimize circadian influences on cardiovascular regulation. Upon arrival, participants rested in the supine position while the experimental procedures were explained and instrumentation for physiological monitoring was applied.

The experimental protocol consisted of three consecutive phases (**Figure 1**): (i) a 30-min baseline period in the supine position; (ii) graded LBNP exposure lasting 16 min; and (iii) a 5-min recovery period in the supine position. LBNP was initiated at −10 mmHg and increased in steps of 10 mmHg every 4 min up to −40 mmHg. This level of negative pressure was selected because it induces central hypovolemia comparable to that occurring during upright posture and is generally well tolerated in healthy individuals (Cooke et al., 2004; Wolthuis et al., 1974; Hinojosa-Laborde et al., 2014; Blaber et al., 2013; Goswami et al., 2008; Cvirn et al., 2019). LBNP was terminated immediately if presyncopal criteria were observed, including marked reductions in arterial blood pressure, light-headedness, visual disturbances, nausea, or at the participant’s request (Gao et al., 2008; Hinghofer-Szalkay et al., 2011; Roessler et al., 2011).

**Figure 1:**
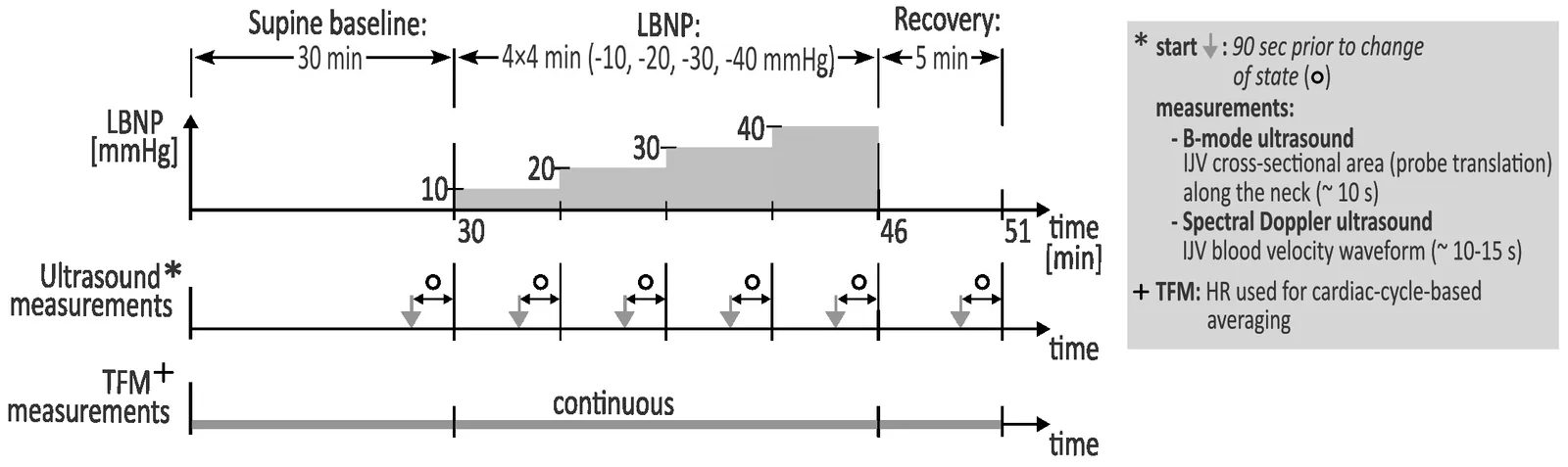
Experimental protocol and timing of measurements. Cardiovascular variables were recorded continuously using a Task Force Monitor (TFM) throughout the experiment. Asterisk (∗) denotes ultrasound acquisition initiation, started approximately 90 s prior to each state transition. Each measurement window included B-mode ultrasound for IJV cross-sectional area (∼10 s probe translation along the neck) and spectral Doppler ultrasound for IJV blood velocity (∼10 s at fixed J2 location). TFM-derived heart rate was used for cardiac-cycle-based averaging of ultrasound signals.

### 2.2 Data acquisition and synchronization

Cardiovascular variables were recorded continuously throughout the protocol using a Task Force^®^ Monitor (TFM 3040i, CNSystems, Graz, Austria), providing beat-to-beat measurements of heart rate, arterial blood pressure, stroke volume, cardiac output, and thoracic impedance. Thoracic fluid content (TFC), derived from the thoracic impedance signal, was used as a non-invasive surrogate index of central (thoracic) blood volume to verify that graded LBNP produced the intended central hypovolemic stimulus. In parallel, ultrasound measurements of the IJV were performed at predefined time points to assess vessel morphology and blood-flow velocity (**Figure 1**). Heart rate data from the TFM were used to synchronize all ultrasound-derived measures to the cardiac cycle. The systemic cardiovascular and autonomic responses of this cohort (heart rate, blood pressure, stroke index, cardiac index, total peripheral resistance index, and heart-rate-variability indices) have been reported previously (Shankhwar et al., 2023) and are not re-analyzed here.

Ultrasound measurements of the IJV were performed at discrete time points during supine baseline, graded LBNP (−10 to −40 mmHg in 4-min steps), and recovery. Within each 4-min stage, hemodynamic responses were first allowed to stabilize, and IJV ultrasound acquisition was then initiated approximately 90 s prior to the change to the next experimental condition (**Figure 1**). For each condition, a B-mode ultrasound recording (∼10 s) was acquired first to determine IJV cross-sectional area during controlled probe translation, waveform at the fixed J2 location. TFM-derived heart rate was used for cardiac-cycle-based processing of both ultrasound-derived area and velocity data.

#### 2.2.1 Jugular vein morphology

Ultrasound imaging of the IJV was performed approximately 90 s prior to each change in experimental condition, after stabilization of hemodynamic responses (as detailed above and in **Figure 1**). Imaging was conducted using a Philips CX50 ultrasound system (Philips Healthcare, Andover, MA, USA) equipped with a Philips L12–3 broadband linear array transducer. Minimal probe pressure was applied to avoid mechanical compression of the vein, and lumen patency was confirmed visually on the B-mode image during each acquisition.

For each experimental condition, approximately 10-s B-mode video clips of the IJV transverse cross-section were acquired. To minimize sensitivity to local anatomical variability and probe positioning, the transducer was translated at a slow and approximately constant speed from the mandibular angle toward the jugulum, as illustrated in **Figure 2A**. Six B-mode clips (one per condition) were recorded for each participant.

**Figure 2:**
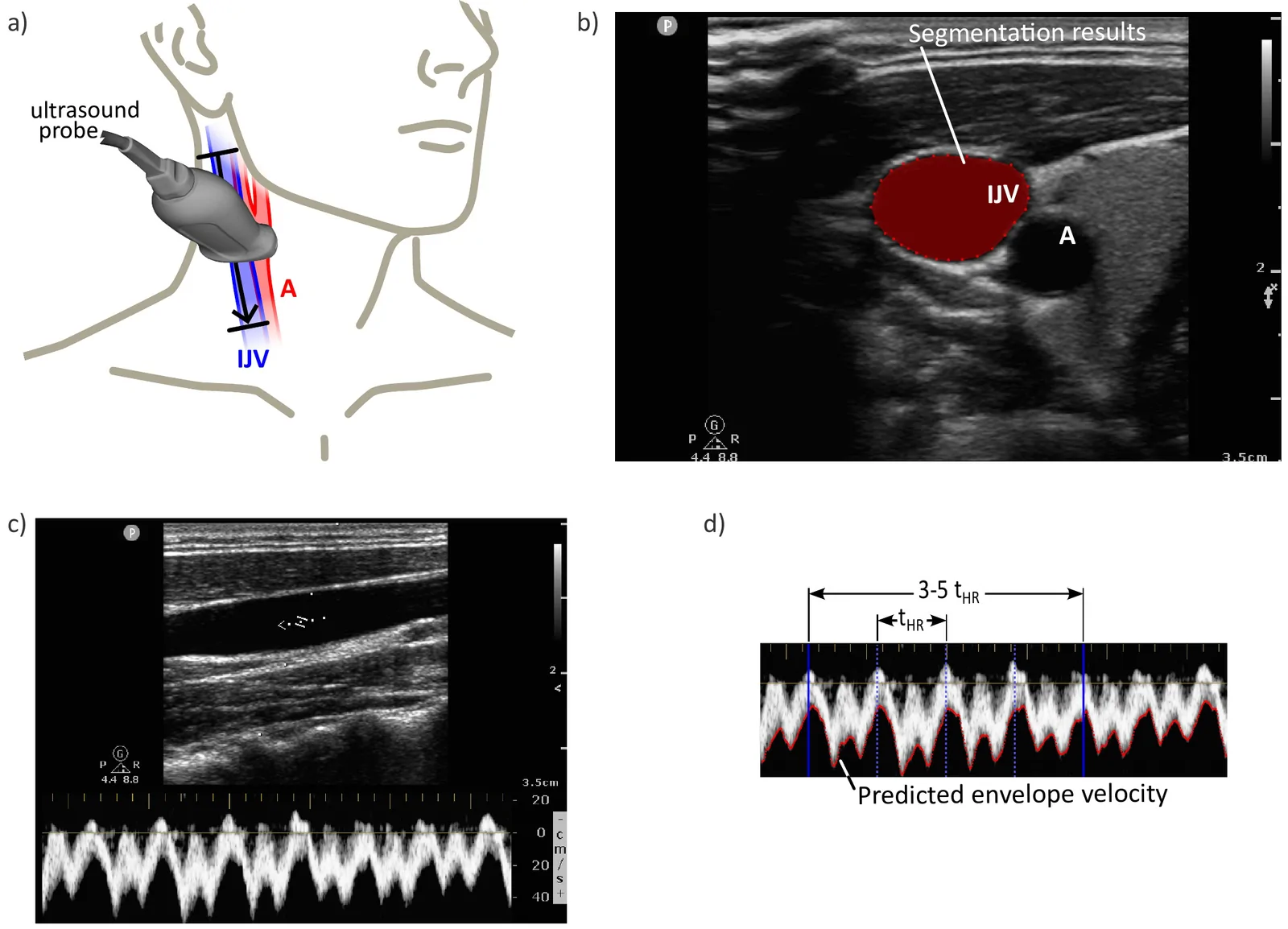
Ultrasound-based assessment of IJV morphology and blood velocity. **(A)** Schematic illustration of ultrasound probe positioning and controlled probe translation along the neck for assessment of IJV cross-sectional area. **(B)** Representative B-mode ultrasound image showing semi-automatic segmentation of the IJV transverse cross-section. **(C)** Example of pulsed-wave spectral Doppler ultrasound recording of IJV blood velocity. **(D)** Doppler-derived velocity envelope averaged over 3–5 cardiac cycles, with cardiac timing defined using synchronized Task Force Monitor heart rate data.

Segmentation and cross-sectional area estimation: The acquired B-mode ultrasound clips were processed using a custom-developed interactive image-analysis tool to determine the IJV cross-sectional area. Segmentation was initiated by manually placing a seed point within the vein lumen on a reference frame. From this point, radial rays were cast in all directions and the lumen border was identified, in each direction, as the first edge detected on the intensity-thresholded image; the resulting boundary points were low-pass filtered to a smooth closed contour, whose centroid was updated and propagated across consecutive frames. The tool was implemented in Wolfram Mathematica. An interactive workflow was intentionally used in preference to a fully automatic algorithm, because the low wall-to-lumen contrast and the high compliance and continuous deformation of the IJV frequently reduced the robustness of unsupervised segmentation.

Because unsupervised segmentation frequently failed, Many automatically generated contours required manual refinement (owing to respiratory and probe motion, gradual changes in vessel geometry, and local image artifacts); all accepted contours were therefore verified by a single experienced operator on a frame-by-frame basis. Frames in which the vein lumen was incompletely visible, affected by anatomical branching, or corrupted by motion artifacts were excluded from further analysis. A representative example of the segmentation outcome is shown in **Figure 2B**; the full segmentation workflow, including the frame-by-frame contours and the resulting along-neck cross-sectional-area profile, is provided in the **Supplementary Methods** (**Supplementary Figure S1**). Reliable segmentation was feasible only where local vessel morphology and image quality permitted, so valid contours covered a variable, largely central portion of each sweep (on average 62% of frames); per-condition segmentation coverage is summarized in **Supplementary Table S4**. A formal quantitative intra-or inter-observer reproducibility assessment was not undertaken and is acknowledged as a limitation.

Reduction of the along-neck area profile to a condition-level mean: Because the transducer was translated along the neck during acquisition, the frame-by-frame segmentation yields a spatial profile *A*(*s*) describing the cross-sectional area as a function of longitudinal position *s* along the neck, rather than repeated measurements of a single fixed cross-section. To attenuate cardiac-phase-related fluctuations, a moving window of length equal to one cardiac cycle (duration defined individually for each condition from TFM-derived heart rate) was translated along this spatial profile, yielding a cardiac-cycle-corrected spatial area profile; the condition-level mean IJV cross-sectional area was calculated as the average of this corrected profile. The cross-sectional area varied substantially along the neck (within-sweep coefficient of variation of *A*(*s*) of approximately 36%; **Supplementary Figure S2**), which is the principal reason area was estimated from a translated sweep rather than from a single plane, since a single-plane measurement would depend critically on the exact probe position.

#### 2.2.2 Jugular vein blood velocity data and volumetric blood flow

Following B-mode acquisition, pulsed-wave spectral Doppler ultrasound was performed at a fixed location (mid-neck, J2 segment; defined as the region of the right IJV at the level of the cricoid cartilage, between the two heads of the sternocleidomastoid muscle) of the internal jugular vein to assess blood flow velocity. The Doppler sample volume was placed in the center of the vessel lumen, and the insonation angle was kept below 60° with angle correction applied along the long axis of the vessel. Doppler recordings of approximately 10–15 s duration were acquired for each experimental condition. Participants breathed spontaneously throughout the protocol; respiratory phase was not gated or explicitly controlled during acquisition, and the velocity envelope was averaged over several consecutive cardiac cycles (see below) to attenuate respiratory-phase-dependent variability. A representative Doppler waveform is shown in **Figure 2C**.

Spectral Doppler signals were synchronized with TFM recordings, allowing identification of individual cardiac cycles (**Figure 2D**). The Doppler velocity envelope was extracted using a custom-developed algorithm and averaged over 3–5 consecutive cardiac cycles for each condition. The mean envelope velocity was calculated by integrating the averaged Doppler velocity envelope over the corresponding cardiac cycles. All Doppler-derived velocity waveforms were visually inspected to confirm physiological plausibility.

Volumetric IJV blood flow was estimated as the product of the mean IJV cross-sectional area and the mean envelope velocity for each experimental condition. This approach assumes that the J2 segment is representative of the mean cross-sectional geometry across the neck region surveyed by the translating probe. Although this introduces a degree of spatial mismatch between the area measurement (spatially averaged along the neck) and the velocity measurement (fixed at J2), the probe-translation approach reduces sensitivity to local anatomical heterogeneity and probe-positioning errors at any single cross-section. The along-neck area profiles and the corresponding spatial-variability analysis that support this averaging approach are provided in the **Supplementary Methods** (**Supplementary Figure S2**; **Supplementary Table S4**). Volumetric flow served as the primary venous hemodynamic outcome variable in subsequent analyses.

Individual IJV cross-sectional area, blood velocity, and volumetric flow data for all participants and experimental conditions are provided in **Supplementary Tables 5**-**S7**.

### 2.3 Statistical analysis

Data were analyzed using linear mixed-effects models to account for the repeated-measures structure and inter-individual variability. Experimental State (Baseline, LBNP10, LBNP20, LBNP30, LBNP40, Recovery) and Sex (Female, Male) were specified as fixed effects, including their interaction, with participant included as a random intercept. Models were fitted using restricted maximum likelihood estimation. Fixed effects were evaluated using Wald tests, and estimated effects are reported as regression coefficients (*β*) with corresponding standard errors (SE).

Female participants served as the reference sex category; consequently, State coefficients represent the LBNP-induced change from baseline within the female group, while State × Sex[Male] interaction terms quantify any additional difference in male responses relative to females. Male-specific effects from baseline were additionally assessed by computing linear contrasts (State + State × Sex[Male]) with corresponding standard errors derived from the model covariance matrix.

To account for potential inter-individual differences in sensitivity to central hypovolemia, an alternative linear mixed-effects model including both random intercepts and random slopes for State within participant was evaluated. Model comparison using likelihood ratio tests and information criteria (AIC) indicated that inclusion of random slopes did not significantly improve model fit. Therefore, results from the more parsimonious random-intercept model are reported.

Because volumetric flow values exhibited right-skewness in the relative-change domain, the response variable was transformed prior to analysis. For each participant, flow was expressed as a relative change with respect to individual supine baseline (Δ*V* = *V*_state_*/V*_baseline_ − 1, where Δ*V* = 0 at baseline). The relative change was then log-ratio transformed using log(1 + Δ*V)*, which is equivalent to modeling the natural logarithm of the flow ratio (log[*V*_state_*/V*_baseline_]). Model coefficients on this scale can therefore be approximately back-transformed to proportional changes via exp(*β*) − 1.

Because the baseline-normalized response variable removes between-sex differences in baseline flow by construction, several complementary analyses were performed to characterize sex effects more completely and to test the robustness of the primary findings. First, an analogous mixed-effects model was fitted to *absolute* log-transformed flow (log*V*state), with State, Sex, and their interaction as fixed effects and participant as a random intercept, so that the Sex main effect reflected the between-sex difference in absolute flow. Second, to distinguish sex-related physiology from body-size effects, baseline IJV cross-sectional area and volumetric flow were additionally indexed to body surface area (BSA) and compared between sexes, consistent with the indexed cardiac parameters reported for this cohort (Shankhwar et al., 2023). Third, because participants with presyncopal termination contribute progressively less data at higher LBNP levels (potentially informative missingness), a sensitivity analysis restricted to participants with complete data at all six states (“completers”) was performed for the relative-change model, and the number of participants with valid flow data was tabulated by state and sex. Finally, TFC was summarized by state and sex to confirm the central hypovolemic stimulus. Given the modest sample size and the exploratory nature of the sex-specific contrasts, effect sizes and 95% confidence intervals are emphasized alongside p-values, and interaction terms are interpreted as hypothesis-generating.

Model assumptions were assessed by visual inspection of residual and quantile–quantile plots to confirm approximate normality and homoscedasticity of residuals. Model residuals did not significantly deviate from normality (Shapiro–Wilk test: W = 0.987, p = 0.050).

Baseline sex differences in participant characteristics and IJV parameters were assessed using Welch’s two-sample t-tests for descriptive purposes only and were not included in the mixed-effects models. Statistical significance was set at p< 0.05. All analyses were performed using Python-based statistical packages (statsmodels, pingouin, scipy, seaborn).

No correction for multiple comparisons was applied, as the analyses were structured around a prespecified primary outcome (volumetric IJV flow). Interaction terms and secondary contrasts should be interpreted with appropriate caution, particularly given the exploratory nature of sex-specific subgroup analyses.

## 3 Results

Participant characteristics are summarized in **Table 1**. As expected, men were significantly taller, heavier, and had a greater body surface area than women (all p< 0.001), and were also slightly older (p = 0.012). No adverse events beyond presyncopal symptoms occurred.

Baseline IJV morphology and hemodynamics are presented in **Table 2**. At baseline, IJV cross-sectional area was larger in men than women (0.851 ± 0.386 vs. 0.598 ± 0.307 cm^2^, p = 0.031) and volumetric flow was higher in men (764.4 ± 396.3 vs. 543 ± 275.6 ml/min, borderline p = 0.052), whereas blood velocity did not differ between sexes (p = 0.643). When indexed to body surface area, neither cross-sectional area (0.445 ± 0.212 vs. 0.366 ± 0.187 cm^2^/m^2^, p = 0.23) nor volumetric flow (401 ± 221 vs. 333 ± 169 ml/min/m^2^, p = 0.29) differed significantly between sexes, indicating that the unindexed baseline differences were largely attributable to body size (see Discussion). These baseline comparisons are descriptive and were not included in the mixed-effects modeling. The effective sample for cross-sectional area and flow analyses comprised 38 participants (18 female, 20 male), after exclusion of participants with insufficient ultrasound image quality or missing baseline data.

**Table 2:** Baseline IJV parameters.

Variable	Sex	Mean ± SD	*n*	p-value (M vs F)
*v*_Jug_ [cm/s]	Female	32.3 ± 14.1	21	0.643
	Male	30.3 ± 12.5	20	
*A*_Jug_ [cm^2^]	Female	0.598 ± 0.307	18	0.031
	Male	0.851 ± 0.386	20	
Φ_Jug_ [ml/min]	Female	543.5 ± 275.6	18	0.052
	Male	764.4 ± 396.3	20	

Values represent baseline (supine) measurements. p-values from Welch’s t-test (two-tailed).

Thoracic fluid content decreased progressively with increasing LBNP in both sexes (by approximately 5% at LBNP40 relative to baseline in females and males alike) and returned toward baseline during recovery, confirming that graded LBNP produced a comparable central hypovolemic stimulus in females and males (**Supplementary Table S2**).

### 3.1 Jugular venous volumetric flow

Jugular venous volumetric flow was analyzed using the linear mixed-effects model described in the Statistical Analysis subsection (female participants as the reference sex category). Model residuals did not deviate significantly from normality (Shapiro–Wilk: W = 0.987, p = 0.050).

Female-specific LBNP effects: Female participants showed significant flow reductions at LBNP30 (*β* = −0.788 ± 0.147, p< 0.001) and LBNP40 (*β* = −1.495 ± 0.175, p< 0.001). No significant reduction was observed at LBNP10 (p = 0.887) or LBNP20 (*β* = −0.241, p = 0.187). Approximately back-transformed, these correspond to a flow reduction of ∼54% at LBNP30 (flow at ∼46% of baseline) and ∼78% at LBNP40 (flow at ∼22% of baseline).

Male-specific LBNP effects: Male participants showed significant flow reductions from LBNP20 onward (LBNP20: Δ = −0.607 ± 0.170, p = 0.0004; LBNP30: Δ = −0.820 ± 0.172, p< 0.001; LBNP40: Δ = −1.001 ± 0.175, p< 0.001). Approximately back-transformed, these correspond to reductions of ∼45% (LBNP20), ∼56% (LBNP30), and ∼63% (LBNP40) from male baseline. No significant change from baseline was detected at LBNP10 (Δ = −0.169, p = 0.320). These male-specific contrast values are derived from the full mixed model (State + State × Sex[Male] terms); the larger apparent female reduction at LBNP40 compared with males (*β*_female_ = −1.495 vs. Δ_male_ = −1.001) corresponds to the non-significant, exploratory state × sex interaction at that level (p = 0.056; see **Figure 3B**; **Supplementary Table S1**).

**Figure 3:**
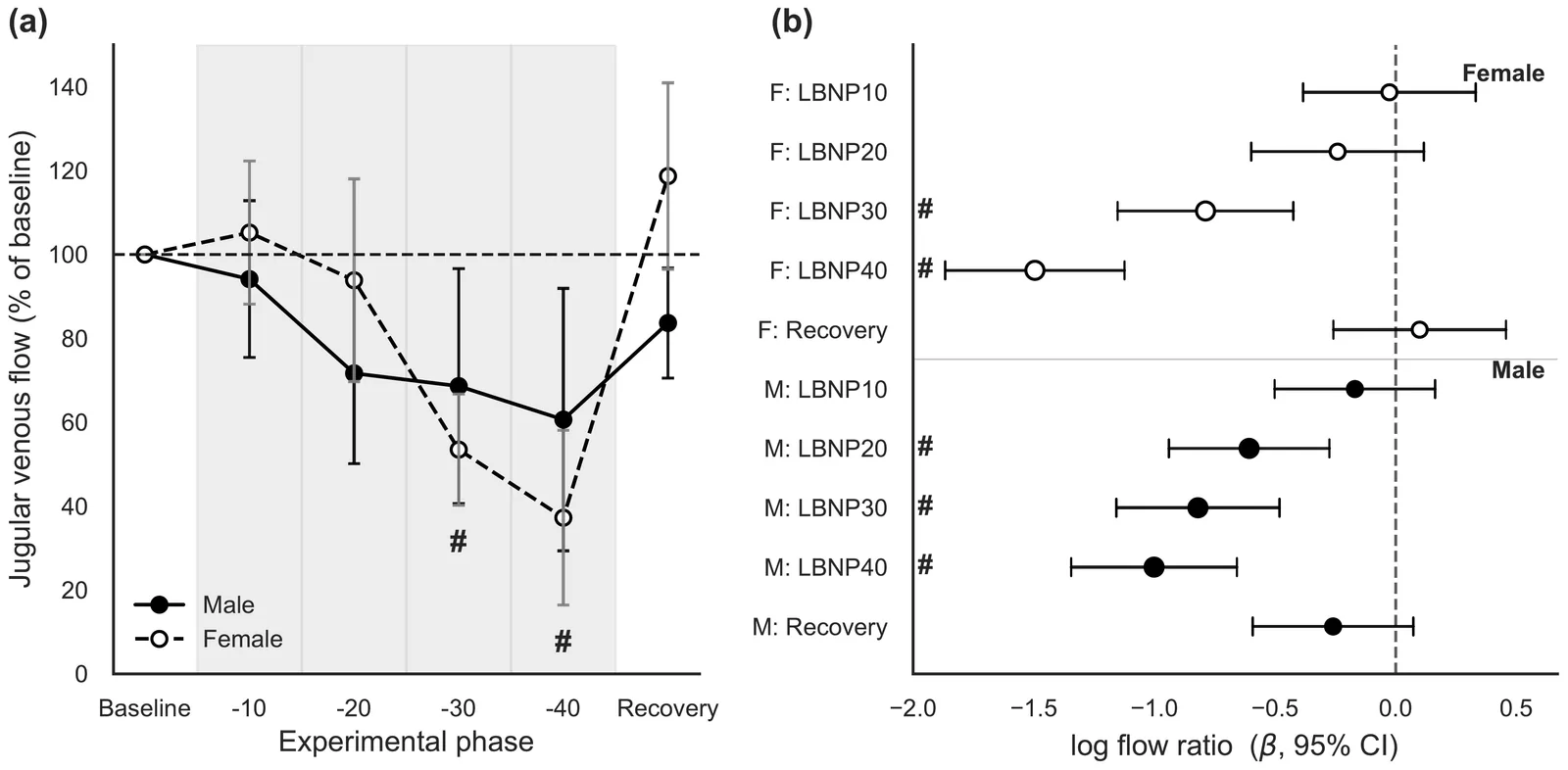
IJV flow response to LBNP. **(A)** Mean ± 95% CI of IJV blood flow changes (expressed as % of baseline) during progressive central hypovolemia (−10 to −40 mmHg LBNP) and recovery. Solid lines with filled markers denote males; dashed lines with open markers denote females. Shaded regions represent active LBNP phases. Both sexes showed progressive flow reductions with increasing LBNP intensity; females demonstrated a numerically larger reduction at LBNP40 (non-significant, exploratory state × sex interaction, p = 0.056). No significant sex difference was observed during the recovery phase (p = 0.150). The hash symbol (#) indicates a statistically significant change from baseline based on the linear mixed-effects model (p< 0.001) for both the female-reference State effect and the male-specific contrast (State + State × Sex[Male]). **(B)** Forest plot of LMM coefficients on the log flow-ratio scale. Open circles show female-reference State effects (*β*± 95% CI); filled circles show male-specific contrasts computed as State + State × Sex[Male]. The vertical dashed line marks no effect (*β* = 0). Intervals entirely to the left of zero indicate a statistically significant flow reduction relative to baseline (p< 0.05, marked by #). Approximate proportional changes follow from exp(*β*) − 1. The full fixed-effect table, including State × Sex[Male] interaction coefficients, is provided in **Supplementary Table S1**. Data are derived from the right IJV.

Sex effects and state × sex interactions: In the primary (baseline-normalized) model, the main effect of sex was not significant (p = 1.00) in males, p = 0.37), while State and interaction terms were essentially unchanged. The state × sex interaction was not statistically significant at LBNP10, LBNP20, or LBNP30 (all p *>* 0.14). A non-significant trend was observed at LBNP40 (State[LBNP40] × Sex[Male]: *β* = +0.494, 95% CI [−0.012, + 0.999], p = 0.056), the positive sign indicating a numerically smaller relative flow reduction in males than females at maximum LBNP and hypothesis-generating. No significant state × sex interaction was observed during Recovery (State[Recovery] × Sex[Male]: *β* = −0.359, 95% CI [−0.847, + 0.130], p = 0.150), indicating that post-LBNP flow restoration did not differ significantly between sexes.

Mean ± 95% CI of IJV blood flow changes during progressive LBNP and recovery are illustrated in **Figure 3A**, together with a forest plot of the female-reference and male-specific LMM contrasts (*β*± 95% CI on the log flow-ratio scale) in **Figure 3B**. The full fixed-effect table from the mixed-effects model, including State × Sex[Male] interaction coefficients, is provided in **Supplementary Table S1**.

## 4 Discussion

The present study demonstrates that progressive lower body negative pressure induces a marked and graded reduction in jugular venous volumetric flow, with significant decreases emerging at moderate to high levels of simulated central hypovolemia. These findings indicate that jugular venous outflow is sensitive to reductions in central blood volume, particularly once compensatory mechanisms are challenged at higher LBNP intensities.

LBNP is a well-established model of central hypovolemia, producing blood pooling in the lower extremities and a reduction in cardiac preload and central venous pressure (Cooke et al., 2004; HinojosaLaborde et al., 2014; Goswami, 2023). The observed reduction in jugular venous flow at higher LBNP levels likely reflects a combination of decreased cerebral venous filling pressure and altered transmural pressure across the jugular vein, leading to partial venous collapse or reduced cross-sectional area. This interpretation is consistent with previous observations that cerebral venous drainage is highly dependent on central venous pressure and thoracic blood volume (Gisolf et al., 2004; Watkins et al., 2017).

Contrary to our *a priori* hypothesis, neither the main effect of sex nor the state × sex interactions across LBNP levels reached significance, indicating that within-participant jugular venous flow responses to graded central hypovolemia were broadly similar between males and females. This similarity held despite known sex differences in autonomic regulation and orthostatic tolerance (Convertino, 1998; Evans et al., 2001; Shankhwar et al., 2023), and despite the significantly different systemic cardiovascular responses documented in the same cohort by Shankhwar et al. (2023), in which females exhibited a greater heart-rate increment and an earlier decline in cardiac index. Notably, thoracic fluid content decreased comparably in both sexes (**Supplementary Table S2**), indicating that the central hypovolemic stimulus was of similar magnitude in females and males; the comparable jugular flow responses therefore cannot be attributed to a weaker Stimulus in either sex. Taken together, these observations are consistent with jugular venous outflow during central hypovolemia beinggoverned predominantly by mechanical factors, specifically reductions in central venous filling pressure and transmural pressure, rather than by sex-dependent autonomic modulation. This interpretation should, however, be regarded as a plausible inference rather than a directly demonstrated mechanism, because intravascular and intracranial pressures were not measured and the systemic autonomic responses were reported separately (Shankhwar et al., 2023).

An exploratory observation of potential interest is the non-significant trend toward a larger relative flow reduction in females at peak LBNP (p = 0.056, 95% CI crossing zero). This trend did not persist in a completers-only sensitivity analysis (p = 0.16), and attrition at higher LBNP levels was modestly greater in females (3 of 18 females vs. 2 of 20 males lacked evaluable flow data at LBNP40; **Supplementary Table S3**). The numerically larger apparent reduction in females at LBNP40 may therefore reflect informative missingness and greater cardiovascular challenge at extreme hypovolemic levels rather than a sex-specific vascular mechanism. This possibilitywarrants investigation in larger samples explicitly powered to detect moderate sex × LBNP interactions.

The baseline sex differences in IJV morphology observed here appear to be largely a function of body size rather than a sex-specific vascular characteristic. Although unindexed cross-sectional area and volumetric flow were larger in males, both differences became non-significant after indexing to body surface area (p = 0.23 and p = 0.29, respectively), consistent with the greater height, mass, and body surface area of the male participants (**Table 1**) and paralleling the body-size-indexed cardiac parameters reported for this cohort (Shankhwar et al., 2023). Baseline IJV morphological differences between sexes should therefore be interpreted as allometric rather than as indicative of a sex-specific venous phenotype.

In contrast to head-up tilt, where gravitational compression of neck veins directly reduces IJV cross-sectional area and produces larger absolute changes in vessel geometry (Ogoh et al., 2022), the LBNP-induced reductions observed here were driven exclusively by reduced central venous filling pressure in the absence of hydrostatic effects. This distinction confirms the utility of supine LBNP for isolating the contribution of intravascular pressure changes from gravitational confounds in studies of cerebral venous hemodynamics.

The graded reduction in jugular venous flow also aligns with autonomic responses typically observed during LBNP, including increased sympathetic activity and altered parasympathetic modulation (Shankhwar et al., 2023; Goswami, 2023). Reduced jugular venous outflow may therefore represent an additional hemodynamic component of the integrative cardiovascular response to central hypovolemia, potentially contributing to changes in intracranial venous pressure and cerebral blood volume regulation during orthostatic stress (Watkins et al., 2017; Ogoh et al., 2022).

Taken together, these findings support the concept that jugular venous drainage is dynamically regulated during central hypovolemia and becomes progressively compromised as hypovolemic stress increases. The preservation of jugular venous flow at mild LBNP, followed by substantial reductions at higher levels, suggests the presence of compensatory mechanisms that maintain jugular venous outflow until a critical threshold of central blood volume depletion is reached (Arbeille et al., 2021; Marshall-Goebel et al., 2019).

The relevance of these findings to spaceflight should be interpreted with appropriate caution. Unlike microgravity, which is characterized by sustained cephalad fluid shifts and an increase in central blood volume, graded LBNP induces central hypovolemia. Accordingly, LBNP is not a model of the microgravity state itself, but rather of the caudal fluid redistribution used as an in-flight and pre-/post-flight countermeasure, and of the orthostatic stress encountered during re-entry and after landing (Arbeille et al., 2021; Marshall-Goebel et al., 2019; Harris et al., 2022). The present findings therefore inform the design and interpretation of LBNP-based countermeasures rather than cerebral venous physiology during weightlessness. Complementary volume-loading models, such as head-down tilt or saline infusion, would be required to characterize the full range of jugular venous responses across both directions of central volume change.

The potential clinical application of ultrasound-derived jugular venous indices for assessment of intravascular volume should likewise be interpreted with caution. Recent point-of-care ultrasound work suggests that jugular venous measurements provide useful but limited information regarding volume status: Aykut et al. (2025), in a fasting-induced dehydration model, found that internal jugular vein distensibility with passive leg raise distinguished dehydrated from control participants but with only modest diagnostic accuracy, whereas cardiac output and velocity-time integral responses performed better. The present study was performed in healthy young volunteers under controlled laboratory conditions and therefore represents a physiological investigation rather than a clinical validation study; extrapolation of these findings to patients with hemorrhage, dehydration, or other forms of circulatory compromise requires further validation in relevant clinical populations.

### 4.1 Limitations

Several limitations should be acknowledged. First, measurements were limited to the right IJV and did not include.

The vertebral venous plexus or the contralateral IJV. Because cerebral venous drainage can redistribute between the jugular and vertebral systems, a reduction in right IJV flow does not necessarily represent an equivalent reduction in total cerebral venous outflow; part of the observed decrease may reflect redistribution rather than a net decline in drainage. This is why we refer to jugular venous outflow, rather than cerebral venous outflow, when describing the present measurements, and total cerebral venous drainage was not quantified. Inter-individual differences and asymmetries in jugular dominance further limit generalization from a single-vessel measurement. Second, jugular venous flow was estimated by combining a spatially averaged cross-sectional area with blood velocity measured at the fixed J2 location. This spatial mismatch, together with the assumption that hemodynamic conditions remained stable during the sequential B-mode and Doppler acquisitions, represents a source of measurement uncertainty. In addition, probe pressure was controlled visually rather than by an instrumented force sensor, and formal intra-or inter-observer reproducibility of the segmentation and flow estimation was not assessed. Third, respiratory influences on jugular venous flow were not controlled. Respiratory-phase-dependent fluctuations in intrathoracic pressure modulate venous return and may contribute to variability in the flow measurements, although averaging over several cardiac cycles was used to attenuate this effect. Fourth, direct intracranial or central venous pressures were not measured, limiting the ability to fully characterize venous outflow gradients, and the mechanistic interpretation therefore relies in part on the systemic and autonomic data reported separately for this cohort (Shankhwar et al., 2023). Fifth, the sample size was modest and the sex-specific contrasts were exploratory; the study was not specifically powered to detect moderate sex × LBNP interactions, and these analyses should be confirmed in larger, adequately powered cohorts.

## 5 Conclusions and future directions

This study demonstrates that graded lower body negative pressure induces a progressive, dose-dependent reduction in jugular venous blood flow, with significant decreases emerging from moderate (−20 to −30 mmHg) LBNP onward. Both male and female participants showed significant reductions at LBNP30 and LBNP40, while males additionally showed a significant reduction at LBNP20. Although males and females exhibit well-established differences in systemic cardiovascular and autonomic responses to central hypovolemia, the jugular venous flow response was remarkably similar between sexes: no significant main effect of sex or state × sex interaction was detected, and baseline morphological differences were largely attributable to body size. These findings indicate that jugular venous outflow during central hypovolemia is governed predominantly by mechanical factors, specifically reductions in central venous filling pressure and transmural pressure, rather than by sex-specific autonomic modulation. This mechanistic interpretation remains to be confirmed with direct intravascular and intracranial pressure measurements, and a non-significant, exploratory trend toward a larger flow reduction in females at peak LBNP (p = 0.056), which did not persist in a completers-only sensitivity analysis, warrants confirmation in larger, adequately powered studies. These findings advance the physiological understanding of jugular venous hemodynamics during central hypovolemia and are relevant to the design and interpretation of LBNP-based countermeasures against spaceflight-related fluid shifts and to the assessment of orthostatic intolerance.

Several complementary lines of inquiry are needed to extend the present observations. First, the extent to which the observed reductions in IJV flow during central hypovolemia translate to changes in optic nerve sheath diameter and retinal microvascular tone remains to be characterized; supine LBNP in the same cohort has already documented sex-specific retinal microcirculatory responses (Saloň et al., 2023b), which under microgravity analogs extend to sustained venular remodeling (Saloň et al., 2023a). Ongoing work in our group is addressing the coupling between jugular, retinal, and optic nerve sheath measures. Second, how IJV flow changes propagate to splanchnic and portal venous circulation during central hypovolemia merits systematic investigation; concurrent reductions in jugular and portal vein volumes during spaceflight (Arbeille et al., 2021) suggest coordinated mechanical coupling across vascular beds. Third, mathematical modeling of the cardiovascular response to LBNP, stratified by sex and incorporating the coupling observed in the present cohort and in related analyses of the same participants (Shankhwar et al., 2023, 2024), may allow prediction of individual tolerance thresholds. Together, such efforts will clarify whether the mechanical determinants identified here for jugular venous outflow generalize to other vascular territories under central hypovolemia.

## Data Availability

The original contributions presented in the study are included in the article/**Supplementary Material**. Further inquiries can be directed to the corresponding authors.
