# Supplementary material for: Internal jugular venous flow responses to graded lower body negative pressure: a sex-stratified analysis
Source: Front Physiol. 2026 Sep 15;17:1873225. doi: 10.3389/fphys.2026.1873225 (PMC13619483; doi:10.3389/fphys.2026.1873225)
Supplement: Supplementary file 1 [file DataSheet1.pdf]

frontiers

Supplementary Material

Bergauer *et al.* — Internal Jugular Venous Flow Responses to Graded Lower Body Negative Pressure: A Sex-Stratified Analysis

SUPPLEMENTARY METHODS

Individual jugular vein haemodynamic data underlying the analyses presented in the main manuscript are provided in the Supplementary Tables. The reported values represent condition-specific mean estimates derived from ultrasound-based measurements of jugular vein cross-sectional area and blood velocity, as described in the Methods section. For each participant and experimental condition, mean internal jugular vein (IJV) blood velocity was obtained from pulsed-wave spectral Doppler recordings averaged over 3–5 consecutive cardiac cycles, and volumetric blood flow was calculated as the product of the mean cross-sectional area and the mean envelope velocity. The estimation of the mean cross-sectional area is described in detail below.

Image segmentation of the jugular vein

The IJV lumen was segmented frame-by-frame from each transverse B-mode cine loop using a custom interactive image-analysis tool implemented in Wolfram Mathematica. For each clip, segmentation was initialized by placing a seed point inside the vein lumen on a reference frame. Radial rays were then cast from this point, and the lumen border was identified in each direction as the first edge detected on the intensity-thresholded image. The resulting boundary points were low-pass filtered to generate a smooth closed contour, whose centroid was subsequently updated and propagated across successive frames.

An interactive workflow was intentionally adopted in preference to a fully automatic segmentation approach because the low wall-to-lumen contrast, high compliance, and continuous deformation of the internal jugular vein during probe translation frequently reduced the robustness of unsupervised segmentation. Consequently, many automatically generated contours required manual refinement owing to respiratory motion, probe motion, gradual changes in vessel geometry, or local image artefacts. All accepted contours were therefore visually verified by the operator on a frame-by-frame basis.

Frames in which the lumen could not be confidently delineated (e.g., because of local vessel branching, out-of-plane motion, or insufficient image quality) were marked as invalid and excluded from subsequent analysis. Segmentation was performed by a single experienced operator. A formal quantitative assessment of intra- or inter-observer reproducibility was not undertaken and is acknowledged as a limitation in the main manuscript.

Because the transducer was translated at an approximately constant speed from the mandibular angle toward the jugulum during each 10 s acquisition, successive frames sampled successive transverse cross-sections of the vein. Consequently, the frame-by-frame area measurements constitute a spatial profile, $A(s)$, describing the cross-sectional area as a function of longitudinal position s along the neck, rather than repeated measurements of a single fixed cross-section. A representative probe sweep, together with the corresponding frame-by-frame segmentation results and the resulting $A(s)$ profile, is shown in Figure S1.

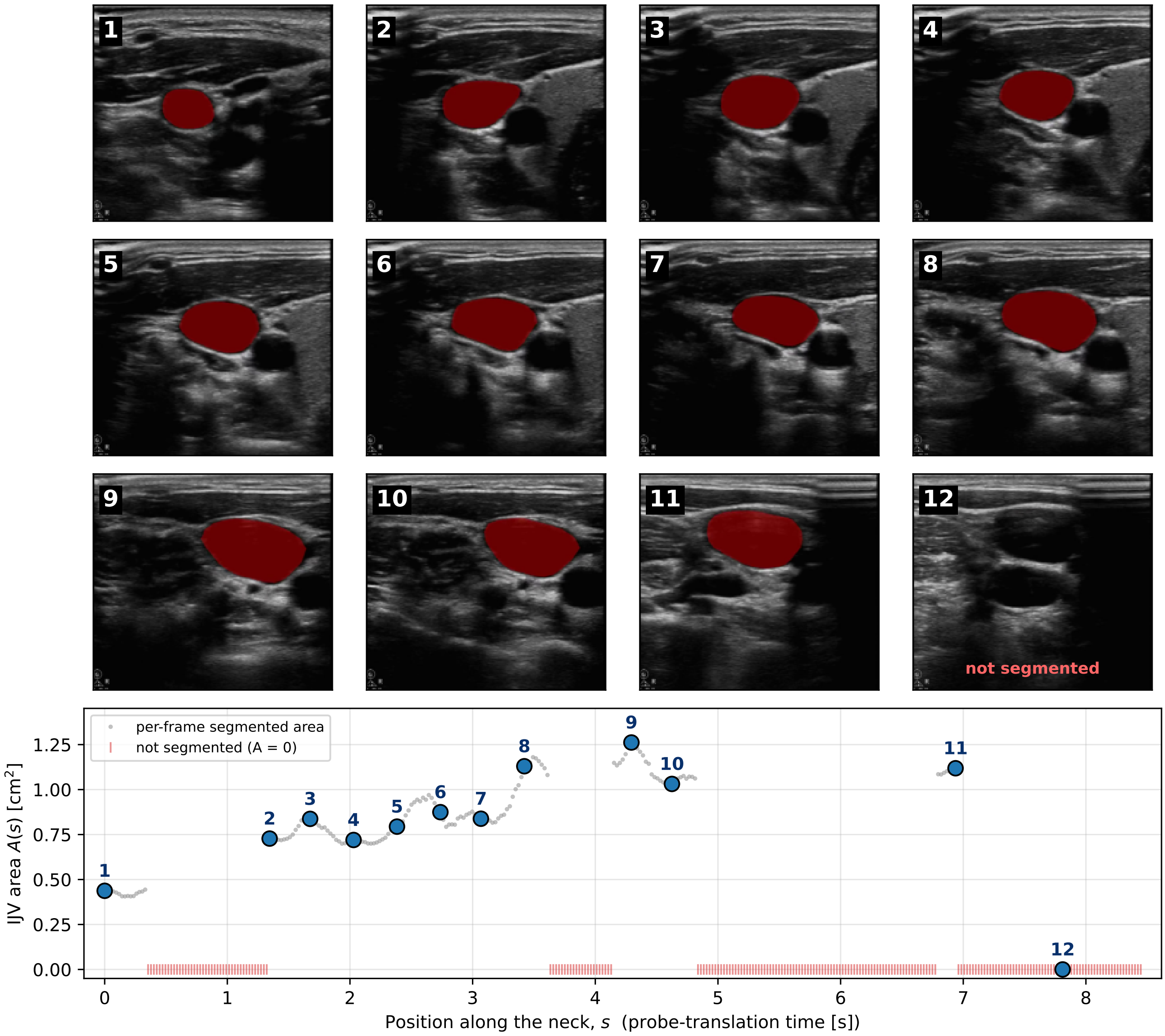

Figure S1. Segmentation workflow for a representative participant and experimental condition (Participant 14, Baseline). Panels 1–12 show B-mode frames sampled at successive positions along the neck during the transverse probe sweep. The red overlay indicates the IJV lumen segmentation produced by the interactive Mathematica-based analysis tool. Panel 12, acquired near the end of the sweep, illustrates a region where the vessel became indistinct and could not be reliably delineated; accordingly, this frame was classified as invalid and excluded from the analysis. The lower panel shows the corresponding cross-sectional-area profile, $A(s)$, obtained from the frame-by-frame segmentation along the neck. The numbered markers indicate the positions of the twelve example frames, whereas red ticks on the horizontal axis denote invalid frames that were excluded from the calculation of the spatially averaged cross-sectional area.

Reduction of $A(s)$ to a condition-level mean area

Each $A(s)$ profile was reduced to a single mean cross-sectional area for each experimental condition. A moving window with a duration equal to one cardiac cycle was translated along the spatial profile, with the cardiac-cycle duration determined from the Task Force Monitor heart rate recorded for the corresponding condition. Windows containing fewer than two-thirds valid segmentations, or whose central frame was

invalid, were discarded. The mean cross-sectional area within each retained window yielded a cardiac-cycle-corrected spatial area profile (solid lines in Figure S2), and the reported condition-level IJV cross-sectional area was calculated as the average of this corrected profile.

Segmentation coverage and spatial variability

Valid segmentation covered a variable fraction of each neck sweep rather than its full length (Table S4). Across the 220 analysed clips, an average of 413 frames per clip (approximately 10.7 s) yielded valid segmentations in 62% of frames (median 64%, range 8–92%). Invalid frames were concentrated predominantly at the proximal and distal ends of the sweep, whereas interior gaps accounted for only approximately 14% of the segmented span. Consequently, each condition-level mean represents a largely contiguous central portion of the scanned vessel.

The cross-sectional area varied substantially along the neck (Figure S2, Table S4). This pronounced spatial variability is the primary rationale for estimating cross-sectional area from a translated probe sweep rather than from a single imaging plane. Because vessel calibre changes considerably along its course, measurements obtained at a single location are highly sensitive to the exact probe position, whereas a spatially averaged estimate is substantially more robust to local anatomical variability and small differences in probe placement.

The same approach also gives rise to the spatial mismatch, acknowledged in the main manuscript, between the spatially averaged cross-sectional area and the blood velocity, which was measured at the fixed mid-neck (J2) location. Consequently, volumetric flow (cross-sectional area $\times$ mean envelope velocity) should be interpreted as a condition-level estimate of jugular venous flow rather than as a plane-matched measurement.

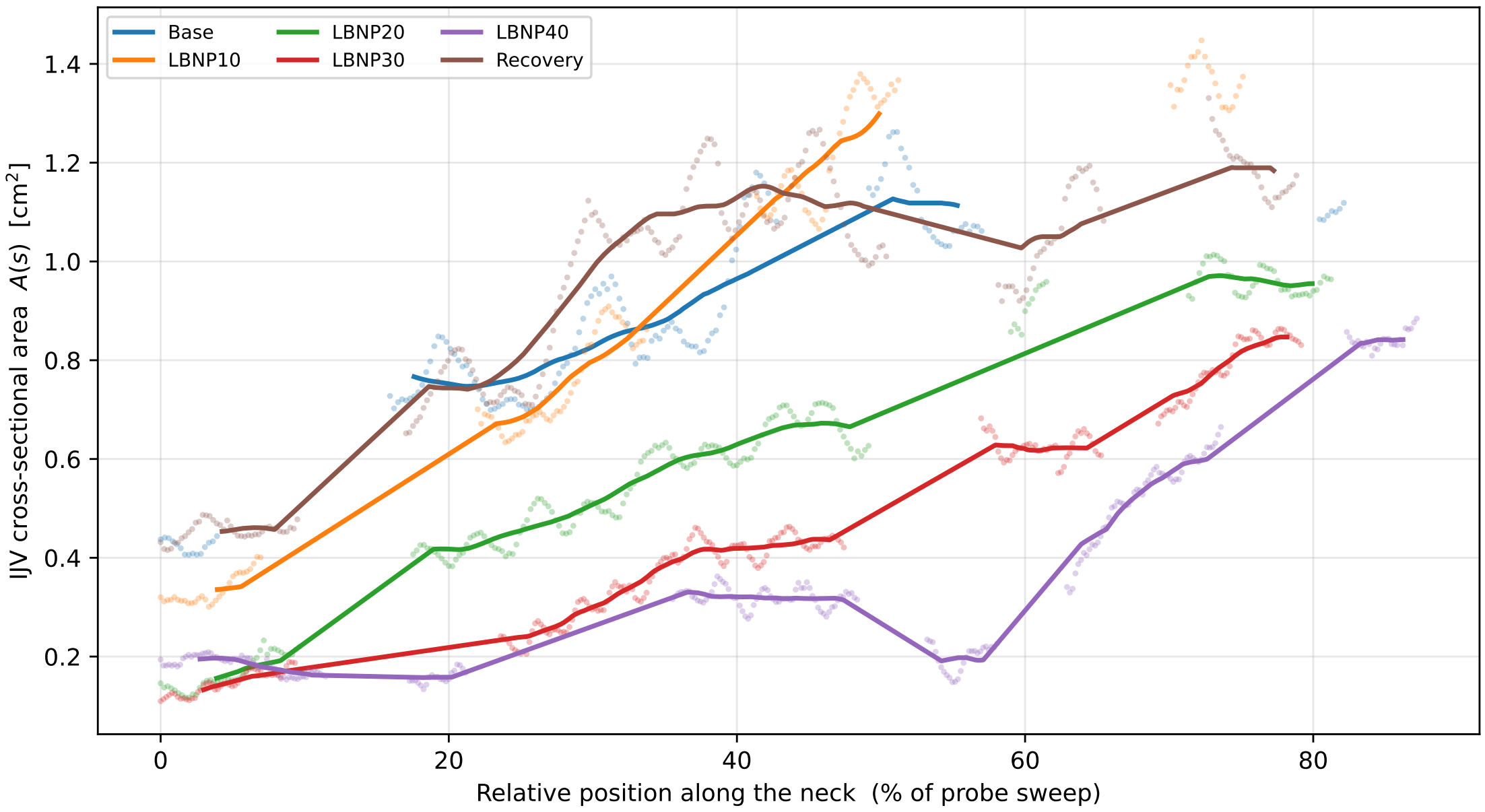

Figure S2. Representative internal jugular vein cross-sectional area profiles, $A(s)$, along the neck for a single participant under all six experimental conditions. The horizontal axis is the relative position along the neck, expressed as the percentage of each probe sweep (from the mandibular angle toward the jugulum); it serves as a position proxy s and is not anatomically calibrated. Normalization to sweep fraction removes between-acquisition differences in absolute sweep duration and speed, so the profiles are comparable across conditions. Faint points denote the frame-by-frame cross-sectional areas obtained from the validated segmentations, whereas the solid lines show the corresponding cardiac-cycle-corrected spatial area profiles used to calculate the condition-level mean cross-sectional area. The figure illustrates both the pronounced spatial variability in vessel calibre along the neck and the progressive reduction in cross-sectional area with increasing lower body negative pressure.

SUPPLEMENTARY TABLES

The main text (Results and Fig. 3b) summarizes the key linear mixed-effects (LMM) coefficients for the female reference group and the male-specific contrasts. Table S1 provides the complete fixed-effect estimates from the LMM, including all State × Sex[Male] interaction coefficients and the random intercept variance.

Table S1. Fixed-effect estimates from the linear mixed-effects model for jugular venous volumetric flow. The response variable is the log-ratio transformed relative change from individual baseline. State coefficients represent the effect of LBNP within the **female** reference group; State × Sex[Male] interactions represent additional differences for males. Wald test p-values; significant effects in bold ($p < 0.05$).

Fixed Effect	Estimate (β)	p-value
Intercept	$+0.000$	1.000
State [LBNP10]	-0.026	0.887
State [LBNP20]	-0.241	0.187
**State [LBNP30]**	$\mathbf{-0.788}$	$\mathbf{< 0.001}$
**State [LBNP40]**	$\mathbf{-1.495}$	$\mathbf{< 0.001}$
State [Recovery]	$+0.099$	0.586
Sex [Male]	0.000	1.000
State [LBNP10] × Sex [Male]	-0.143	0.566
State [LBNP20] × Sex [Male]	-0.367	0.141
State [LBNP30] × Sex [Male]	-0.032	0.901
State [LBNP40] × Sex [Male]	$+0.494$	0.056
State [Recovery] × Sex [Male]	-0.359	0.150
Group Var (random intercept)	0.563	0.003

Model: $\log(V_{\text{state}}/V_{\text{baseline}}) \sim \text{State} \times \text{Sex} + (1 \mid \text{Subject})$.
Note on coefficients: State[x] = female-specific LBNP effect; State[x] × Sex[Male] = additional male deviation. Male-specific contrasts (State + State × Sex[Male]) are reported separately in the main-text Results and visualized in Fig. 3b. Approximate proportional flow changes can be obtained as $\exp(\beta) - 1$.

Thoracic fluid content (TFC), derived from the thoracic impedance signal of the Task Force Monitor, was used as a non-invasive surrogate index of central (thoracic) blood volume to verify that graded LBNP produced the intended central hypovolemic stimulus. Table S2 reports the group mean ± SD TFC by experimental state and sex, together with the mean relative change from baseline. TFC decreased progressively with increasing LBNP in both sexes and returned toward baseline during recovery, with a comparable magnitude of change in females and males (approximately -5% at LBNP40 in both), indicating that the central hypovolemic stimulus was of similar magnitude in the two groups.

Table S2. Thoracic fluid content (TFC; arbitrary units, $1/\text{k}\Omega$) by experimental state and sex, with mean relative change from baseline ($\Delta\%$). Values are mean ± SD; n = number of participants with valid TFC data.

	Female		Male	
State	Mean ± SD (n)	$\Delta\%$	Mean ± SD (n)	$\Delta\%$
Baseline	42.40 ± 5.13 (21)	—	44.97 ± 6.75 (21)	—
LBNP10	41.66 ± 5.18 (20)	−1.1	44.45 ± 6.84 (21)	−1.2
LBNP20	41.00 ± 5.14 (20)	−2.7	43.86 ± 6.82 (21)	−2.5
LBNP30	39.93 ± 5.08 (19)	−4.6	43.11 ± 6.44 (22)	−4.1
LBNP40	39.50 ± 4.78 (19)	−5.5	42.84 ± 6.43 (22)	−4.8
Recovery	42.18 ± 5.30 (21)	−0.6	44.85 ± 6.77 (22)	−0.3

Note: TFC is reported in the native Task Force Monitor units ($1/\text{k}\Omega$); higher values indicate greater thoracic fluid content. Relative change is computed per participant with respect to individual baseline and averaged within each sex.

Table S3 reports the number of participants with valid volumetric-flow data at each experimental state, stratified by sex, within the analytical sample (18 female, 20 male). The declining counts at higher LBNP levels reflect early termination of LBNP exposure due to presyncopal criteria and occasional technical exclusions; because presyncopal termination is physiologically related to tolerance of central hypovolemia, this attrition may not be completely at random and is addressed by the completers-only sensitivity analysis reported in the main text.

Table S3. Number of participants with valid IJV volumetric-flow data by experimental state and sex.

State	Female (n)	Male (n)
Baseline	18	20
LBNP10	17	20
LBNP20	17	20
LBNP30	16	19
LBNP40	15	18
Recovery	17	20

Note: Analytical sample of 38 participants (18 female, 20 male) with valid baseline flow. Completers with valid data at all six states: $n = 31$ (14 female, 17 male).

Table S4 summarizes the quality-control characteristics of the B-mode segmentation across all analysed clips: the number of clips per state, the mean number of frames and sweep duration, the fraction of frames with a valid lumen segmentation (coverage), and the within-clip spatial variability of the cross-sectional area along the neck.

Table S4. Segmentation quality-control summary by experimental state. Values are means across clips.

State	Clips (n)	Frames/clip	Sweep [s]	Coverage [%]	CV of $A(s)$ [%]
Baseline	38	391	9.8	57.8	27.3
LBNP10	38	401	10.5	61.7	32.2
LBNP20	37	425	11.0	59.3	36.4
LBNP30	37	428	11.2	63.2	42.0
LBNP40	33	412	10.9	64.6	47.8
Recovery	37	423	11.1	65.8	29.9
All	220	413	10.7	62.0	35.6

Note: Coverage = percentage of frames with a valid lumen segmentation. CV of $A(s)$ = within-clip coefficient of variation of the segmented cross-sectional area along the neck (mean across clips). Pooled coverage: median 64.4%, range 8.2–91.9%. Pooled within-clip CV of $A(s)$: median 33.3%, interquartile range 25.8–42.8%.

Table S5 reports individual mean IJV blood velocity values for all participants across experimental conditions, including supine baseline, graded lower body negative pressure (LBNP; -10 to -40 mmHg), and recovery. Missing values (reported as NaN) indicate experimental conditions that were not completed due to early termination of LBNP exposure based on presyncopal criteria or technical issues.

Table S5: Mean internal jugular vein blood velocity [cm/s].

Subject ID	Baseline	LBNP10	LBNP20	LBNP30	LBNP40	Recovery
1	NaN	NaN	NaN	NaN	NaN	NaN
2	18.79	35.83	29.71	14.53	6.25	24.94
3	7.54	14.33	16.92	14.04	19.19	9.06
4	19.89	9.52	10.61	5.15	4.50	11.09
5	8.47	7.15	14.68	14.70	21.17	6.65
6	14.90	5.74	5.49	10.14	8.61	9.44
7	8.26	13.51	15.93	12.62	22.76	8.18
8	27.72	28.41	1.87	2.18	NaN	19.54
9	27.19	41.06	53.83	24.07	21.78	51.05
10	29.27	40.88	46.35	48.96	8.63	30.39
11	NaN	8.19	7.74	12.13	7.77	39.17
12	13.18	11.68	29.55	6.91	3.19	15.66
13	13.97	25.81	41.65	35.72	28.05	31.14
14	16.50	15.51	20.62	34.33	26.69	11.37
15	14.87	17.79	17.30	18.58	16.14	12.32
16	10.26	13.48	9.32	12.19	25.18	13.99
17	NaN	NaN	NaN	NaN	NaN	NaN
18	8.38	11.54	12.96	25.59	45.64	10.67
19	9.75	12.41	21.46	46.11	52.45	12.29
20	21.77	23.37	7.90	3.52	9.29	28.97
21	25.82	32.27	35.06	13.99	7.20	19.42
22	11.73	14.91	9.55	NaN	3.96	12.25
23	6.77	9.31	18.18	11.99	8.09	5.61
24	13.01	NaN	NaN	NaN	NaN	18.79
25	22.67	14.02	30.39	45.23	34.60	7.89
26	15.81	29.91	12.05	27.38	25.32	7.54
27	20.32	10.83	9.65	8.45	4.33	15.82
28	23.08	33.40	23.61	41.28	28.70	32.85
29	10.52	12.40	20.24	12.38	NaN	21.16
30	15.97	19.43	20.56	14.91	12.65	19.20
31	20.13	30.92	37.07	35.82	51.12	40.22
32	15.12	15.06	9.35	8.48	9.68	13.87
33	8.40	5.75	3.32	3.60	NaN	8.44
34	9.11	5.71	5.62	4.14	4.63	6.36
35	18.32	13.69	20.76	16.48	6.40	8.66
36	10.33	17.67	37.20	10.47	18.36	18.73

Continued on next page

Table S5 continued.

Subject ID	Baseline	LBNP10	LBNP20	LBNP30	LBNP40	Recovery
37	23.13	26.53	31.36	39.30	NaN	12.53
38	11.87	16.69	21.25	19.70	12.19	15.20
39	20.50	16.93	11.14	14.30	NaN	20.21
40	12.91	21.17	12.08	19.06	10.86	21.08
41	6.88	8.68	10.11	13.30	4.41	6.98
42	23.89	11.85	14.49	6.97	6.94	27.52
43	20.80	22.55	20.21	27.25	15.82	11.13
44	4.35	3.53	1.62	NaN	1.80	3.57

Table S6 reports individual mean IJV cross-sectional area values for all participants across experimental conditions. Values represent spatially averaged, cardiac-cycle-corrected estimates derived from B-mode ultrasound segmentation (see Supplementary Methods).

Table S6: Mean internal jugular vein cross-sectional area [cm^2].

Subject ID	Baseline	LBNP10	LBNP20	LBNP30	LBNP40	Recovery
1	NaN	NaN	NaN	NaN	NaN	NaN
2	0.510	0.428	0.247	0.207	0.179	0.506
3	1.731	1.373	1.109	1.170	0.674	1.765
4	0.407	0.351	0.229	0.192	0.158	0.260
5	0.910	0.854	0.787	0.623	0.409	0.811
6	0.433	0.357	0.169	0.117	0.214	0.432
7	1.112	0.894	0.842	0.624	0.541	1.278
8	NaN	NaN	NaN	NaN	NaN	NaN
9	0.468	0.320	0.233	0.250	0.164	0.611
10	0.618	0.530	0.495	0.213	0.145	0.630
11	NaN	NaN	NaN	NaN	NaN	NaN
12	0.340	0.358	0.156	0.149	0.122	0.360
13	0.821	0.640	0.495	0.296	0.302	0.657
14	0.880	0.891	0.582	0.458	0.365	0.966
15	0.910	0.892	0.626	0.538	0.521	1.027
16	1.153	1.274	1.175	0.852	0.582	1.011
17	NaN	NaN	NaN	NaN	NaN	NaN
18	1.326	0.924	0.826	0.417	0.246	0.734
19	1.352	1.268	1.073	0.758	0.741	1.311
20	0.547	0.581	0.260	0.154	0.137	0.504
21	1.052	0.779	0.767	0.551	0.268	1.342
22	0.833	0.616	0.424	0.327	0.165	0.797
23	0.536	0.454	0.330	0.167	0.183	0.687
24	0.272	0.226	NaN	NaN	NaN	NaN
25	0.438	0.450	0.271	0.183	0.100	0.488
26	1.128	1.179	0.612	0.426	0.429	1.318
27	0.817	0.529	0.276	0.192	0.198	0.774
28	0.905	0.659	0.379	0.283	0.243	0.697
29	0.277	0.249	0.248	0.069	NaN	0.241
30	1.014	1.262	0.789	0.377	0.336	0.914
31	1.089	0.645	0.687	0.576	0.350	0.624
32	0.404	0.241	0.140	0.097	0.133	0.502
33	0.794	0.784	0.757	0.663	NaN	0.565
34	0.458	0.340	0.404	0.384	0.294	0.337
35	NaN	NaN	NaN	NaN	NaN	NaN
36	NaN	NaN	NaN	NaN	NaN	NaN

Continued on next page

Table S6 continued.

Subject ID	Baseline	LBNP10	LBNP20	LBNP30	LBNP40	Recovery
37	0.744	0.767	0.512	0.460	NaN	0.288
38	1.179	1.055	0.583	0.346	0.246	1.104
39	0.599	0.290	0.226	0.196	NaN	0.473
40	0.399	0.325	0.223	0.117	0.107	0.284
41	0.146	0.111	0.091	0.070	0.058	0.127
42	0.471	0.297	0.200	0.206	0.116	0.416
43	0.587	0.462	0.193	0.193	0.100	0.814
44	0.117	0.106	0.069	0.038	0.110	0.117

Table S7 reports individual mean IJV volumetric blood flow values for all participants across experimental conditions. Volumetric flow was calculated as the product of mean cross-sectional area and mean envelope velocity for each condition.

Table S7: Mean internal jugular vein volumetric blood flow [ml/min].

Subject ID	Baseline	LBNP10	LBNP20	LBNP30	LBNP40	Recovery
1	NaN	NaN	NaN	NaN	NaN	NaN
2	574.8	920.7	440.6	180.5	67.1	757.6
3	783.1	1179.9	1125.7	985.1	776.2	959.0
4	485.6	200.5	146.0	59.3	42.8	173.1
5	462.3	366.6	693.3	549.6	519.5	324.0
6	387.2	123.0	55.6	71.1	110.8	244.5
7	551.3	724.5	804.9	472.9	738.2	627.2
8	NaN	NaN	NaN	NaN	NaN	NaN
9	763.4	788.0	753.5	360.3	214.7	1871.7
10	1086.2	1300.1	1376.0	626.7	75.2	1148.0
11	NaN	NaN	NaN	NaN	NaN	NaN
12	268.5	250.7	276.0	61.6	23.2	338.3
13	688.3	991.3	1236.4	634.8	507.8	1228.1
14	871.2	829.5	720.4	944.1	584.6	659.0
15	811.6	952.5	649.6	599.9	504.3	758.9
16	709.8	1030.5	657.3	623.4	880.1	848.6
17	NaN	NaN	NaN	NaN	NaN	NaN
18	666.8	639.9	642.4	640.5	674.0	469.9
19	791.2	944.5	1381.6	2096.4	2332.3	966.1
20	713.9	815.2	123.3	32.5	76.6	876.1
21	1629.6	1507.5	1612.6	462.5	115.9	1563.9
22	586.2	551.1	242.9	NaN	39.2	585.5
23	217.9	253.4	359.7	119.8	88.9	231.4
24	212.5	NaN	NaN	NaN	NaN	NaN
25	595.2	378.3	494.0	496.3	207.1	231.0
26	1070.6	2115.6	442.4	700.4	651.2	595.9
27	996.4	343.4	159.8	97.4	51.3	734.2
28	1253.2	1320.9	536.6	701.4	418.3	1373.8
29	174.5	184.9	301.4	51.4	NaN	305.5
30	971.6	1471.3	973.8	336.9	254.9	1053.2
31	1314.8	1196.3	1527.4	1237.1	1074.2	1506.0
32	366.7	217.5	78.5	49.1	77.3	417.7
33	400.2	270.5	150.6	143.1	NaN	286.4
34	250.3	116.4	136.3	95.4	81.7	128.6
35	NaN	NaN	NaN	NaN	NaN	NaN
36	NaN	NaN	NaN	NaN	NaN	NaN

Continued on next page

Table S7 continued.

Subject ID	Baseline	LBNP10	LBNP20	LBNP30	LBNP40	Recovery
37	1032.1	1221.4	962.7	1085.3	NaN	216.7
38	839.1	1056.3	743.3	408.9	179.6	1006.5
39	737.2	294.8	151.1	167.9	NaN	573.4
40	309.1	413.4	161.5	133.9	69.6	358.6
41	60.3	57.7	55.0	55.5	15.3	53.1
42	674.7	211.2	173.8	86.0	48.2	687.0
43	733.2	624.7	234.0	316.0	95.0	543.1
44	30.5	22.4	6.7	NaN	11.8	25.0
